# Assessment of Quality and Utility of Patient-Taken Smartphone Photographs of Atopic Dermatitis: Clinical Survey Study

**DOI:** 10.2196/72916

**Published:** 2026-01-27

**Authors:** Zarqa Ali, Kenneth Thomsen, Christian Vestergaard, Simon Francis Thomsen

**Affiliations:** 1 Department of Dermatology, Bispebjerg Hospital, Bispebjerg Bakke 23, Copenhagen, 2400, Denmark, 45 60833238; 2 Department of Dermatology and Venereology, Aarhus University Hospital, Aarhus, Denmark

**Keywords:** atopic dermatitis, photograph, telemedicine, teledermatology, outpatient clinic, personalized, follow-up

## Abstract

**Background:**

Atopic dermatitis (AD) has a relapsing and remitting nature, and scheduled clinic visits only provide a snapshot of the skin condition at the moment.

**Objective:**

This study aimed to investigate the quality of patient-taken smartphone photographs of AD skin lesions and characterize patients using smartphone photographs as a tool to assist the physician to show disease activity in between consultations.

**Methods:**

Patients from 2 university outpatient clinics specialized in AD were surveyed. A questionnaire regarding digital readiness was completed, and a previously taken skin lesion photograph on the patients’ own smartphone was evaluated.

**Results:**

Between February 2024 and September 2024, a total of 100 questionnaires were completed, 60 (60%) by participants from the capital region of Denmark and 40 (40%) by participants from an urban area, including 62 (62%) men and 38 (38%) women. The mean age of the recruited patients was 33.9 (SD 19.9) years. A total of 78% (78/100) of the patients used a desktop computer, laptop, or tablet often or always, and 86% (86/100) corresponded with the health care system using technology (eg, via email to the general practitioner or contact with hospitals via apps). More than 50% (52/100, 52%) strongly agreed or agreed with the statement that they would prefer a remote online visit with, for example, upload of skin lesion photographs over a routine in-person office visit. Almost 3 out of 4 patients had a photograph of their AD skin lesion on their smartphone, most (38/71, 54%) with the sole intention of presenting it to a physician. The photographs were of good quality in 85% (60/71) of the cases, and most (61/71, 86%) of the smartphone photographs were assessed to be useful for diagnostic and clinical evaluation. Receiving topical monotherapy was significantly associated with increased risk of having taken a skin lesion smartphone photograph (*P*=.006).

**Conclusions:**

Patients with AD followed up on in an outpatient clinic often took good-quality photographs of their skin lesions before consultations with the intention of presenting them to the physician.

## Introduction

Atopic dermatitis (AD) has a fluctuating nature, including unpredictable flares [1], which is why scheduled visits to an outpatient clinic only provide a momentary snapshot of the disease course. Patients’ perception of the use of photographs of skin lesions in clinical settings to improve medical care is overall positive [2]. A qualitative study highlighted that patients often feel unheard when consulting their physicians in times of disease remission. It also demonstrated an unfulfilled desire to be able to show a flair either by writing down symptoms or photographing lesions during flairs. Patients also indicated that the ability to evaluate the skin in between consultations provides increased autonomy and ownership [3]. A study conducted in an urticaria outpatient clinic showed that patients often took photographs of their skin lesions with their own smartphones before their first consultation, providing the physician with an insight into their disease severity at times of flare [4]. It has also been confirmed that the use of smartphones to take photographs of skin lesions is growing rapidly, a trend that might reduce the need for referrals to face-to-face visits [5] and thereby mitigate the growing shortage of dermatologists [6]. Furthermore, the severity of AD can be reliably assessed using photographs taken using smartphones as there is a high agreement between assessments conducted in the clinic directly looking at the skin and assessments conducted based on photographs [7,8].

Due to the clearly visible morphology of AD and the growing use of photographs taken using smartphones for medical documentation purposes, we aimed to investigate the quality of patient-taken photographs of AD skin lesions using smartphones. Second, we aimed to characterize the group of patients who take smartphone photographs as a tool to assist the physician’s evaluation of disease activity in between consultations. This knowledge might help understand patient preferences and tailor an individualized plan for follow-up either face-to-face or remotely based on photographs, thereby reducing health care costs while increasing patient autonomy.

## Methods

### Overview

Patients were consecutively recruited from 2 university outpatient clinics specialized in AD; one clinic in the capital region of Copenhagen and one from the second-largest urban area in Denmark, Aarhus. From February 2024 to September 2024, patients with a consultation in one of the outpatient clinics were asked to complete a questionnaire and select a possible previously taken smartphone photograph of their own AD lesions for severity assessment and quality evaluation by the physician. For pediatric patients, the questionnaire was completed by the parents.

To measure the perception of the impact of AD on quality of life, the Skindex-Mini, a 3-item questionnaire assessing 3 domains (symptoms, emotions, and function) graded on a Likert scale from 0 to 6, was used [9]. The Skindex-Mini total score was used to stratify impact of skin conditions on patient’s quality of life as follows: a score of 0 to 1 indicated no impact, a score of 2 to 5 indicated low impact, a score of 6 to 10 indicated moderate impact, a score of 11 to 14 indicated high impact, and a score of 15 to 18 indicated very high impact on quality of life. The questionnaire has also been validated in pediatric patients with AD [10]. Questions related to use of technology in general and for communication with health care professionals were also included [11].

On the basis of the selected photograph of an AD lesion taken by the patient on their smartphone, a questionnaire regarding the quality and utility of smartphone photographs of AD skin lesions was completed by the attending physician. The quality assessment was based on focus of the photograph, resolution, lighting, and blurriness [12,13]. The utility of smartphone photographs for diagnostic use was based on the overall assessment of the treating physician (ie, whether the treating physician felt confident when using the photograph to establish diagnosis and for clinical evaluation and severity assessment). The clinical signs from the Eczema Area and Severity Index (EASI) and Scoring Atopic Dermatitis (SCORAD), including erythema, edema or papulation, excoriation, lichenification, oozing or crusts, and dryness, were assessed for each photograph along with their intensity (0-3 for the EASI and 0-4 for SCORAD) [14,15].

### Statistical Analysis

Chi-square and independent-sample 2-tailed *t* tests were used to characterize patients who took smartphone photographs of skin lesions, and 95% CIs were provided when applicable. The Fisher exact test was used when one or more of the cells had an expected frequency of 5 or less. Multiple logistic regression was used to identify the variables best related to the likelihood of patients having a smartphone photograph of a skin lesion, including age (<30 years vs >30 years), sex, capital or urban area residence, AD onset (<2 years of age vs >2 years of age), quality of life (Skindex-Mini total score), systemic treatment vs topical treatment, daily use of technology, digital contact with the health care system, and whether they preferred a remote visit. A *P* value of <.05 was considered statistically significant. All tests were carried out using the SPSS software (version 25.0; IBM Corp) [16].

### Ethical Considerations

As this was a questionnaire study, there was no requirement of governmental approval or written informed consent according to Danish guidelines [17]. All study participants gave oral consent to be included in the study and have their data stored. All data used in this study were fully anonymized. No personally identifiable information was collected, stored, or processed, ensuring the privacy and confidentiality of all participants. Participants did not receive any financial or nonfinancial compensation for their participation in this study.

## Results 

### Cohort Description

A total of 100 questionnaires were completed, 60 (60%) by participants from the capital region and 40 (40%) by participants from the urban area, including 62 (62%) men and 38 (38%) women. The median age of the recruited patients was 28.0 (IQR 20.25-48.75; mean age 33.9, SD 19.9) years. Most (n=53, 53%) had an AD onset before the age of 2 years, 25% (n=25) had an AD onset between the ages of 2 and 6 years, 10% (n=10) had an AD onset between the ages of 6 and 18 years, and the remaining 12% (n=12) had an AD onset after the age of 18 years. A total of 37% (n=37) of the patients were treated with topical corticosteroids in monotherapy at the time of consultation, 36% (n=36) were treated with dupilumab, 12% (n=12) were treated with methotrexate, 3% (n=3) were treated with tralokinumab, and 3% (n=3) were treated with baricitinib or abrocitinib. Most patients (n=64, 64%) estimated AD to have none or a small impact on quality of life, 19% (n=19) estimated AD to have a moderate impact, 10% (n=10) estimated AD to have a large impact, and 7% (n=7) estimated AD to have a very large impact based on the Skindex-Mini questionnaire (Table 1).

**Table 1.** Characteristics of the included patients from 2 atopic dermatitis outpatient clinics (N=100).

Characteristic	Values
Sex, n (%)	
Male	62 (62)
Female	38 (38)
Age (years), mean (SD)	33.9 (19.9)
Current treatment, n (%)	
Topical treatment^a^ only	38 (38)
UVB	1 (1)
Traditional immunosuppressants^b^	16 (16)
Prednisolone	1 (1)
JAK^c^ inhibitors	3 (3)
Biologics^d^	39 (39)
None	2 (2)
Skindex-Mini score (0-18), mean (SD)	
Symptoms	2.25 (1.88)
Emotions	1.51 (1.81)
Function	1.30 (1.77)
Total	5.02 (5.03)
Impact on quality of life, n (%)	
None	33 (33)
Small	31 (31)
Moderate	19 (19)
Large	10 (10)
Very large	7 (7)

a Topical corticosteroids and topical calcineurin inhibitors.

b Azathioprine, methotrexate, and mycophenolate mofetil.

c JAK: Janus kinase; inhibitors included abrocitinib and baricitinib.

d Dupilumab and tralokinumab.

### Digital Readiness

In total, 78% (78/100) of the patients used a computer, laptop, or tablet often or always; 18% (18/100) used them seldom or once in a while; and 4% (4/100) never used them. A vast majority (86/100, 86%) corresponded with the health care system using technology (eg, via email to the general practitioner or contact with hospitals via apps). More than 50% (52/100, 52%) strongly agreed or agreed with the statement that they would prefer a remote online visit with, for example, upload of skin lesion photographs over a routine in-person office visit. Table 2 provides further details.

**Table 2.** Items related to attitudes toward digital solutions (N=100).

Digital readiness	Participants, n (%)
Daily use of a computer, laptop, or tablet	
Often or always	78 (78)
Seldom or once in a while	18 (18)
Never	4 (4)
Digital correspondence with the health care system	
Often or always	56 (56)
Seldom or once in a while	30 (30)
Never	14 (14)
Digital access to blood samples or medical records	
Often or always	53 (53)
Seldom or once in a while	34 (34)
Never	13 (13)
Search for information related to morbidity on the internet	
Often or always	42 (42)
Seldom or once in a while	35 (35)
Never	23 (23)
“I would like to replace a physical in-office visit with a remote visit.”	
Strongly agree	19 (19)
Agree	33 (33)
Neutral	27 (27)
Disagree	12 (12)
Strongly disagree	9 (9)

### Smartphone Photographs

Almost 3 out of 4 patients (71/100, 71%) had a photograph of their AD skin lesion on their smartphone. Of the remaining 29% (29/100) who did not have any photographs of their AD lesions on their smartphones, most (15/29, 52%) indicated that the reason was a well-controlled disease for a longer period without experiencing any flair or worsening of AD, only 3% (1/29) did not have a smartphone, 7% (2/29) used another smartphone to take photographs, and the remaining 38% (11/29) did not give a reason. The number of smartphone photographs of AD lesions taken in the previous year varied from 1 to 100, the mean number of photographs taken was 21.4 (SD 22.7), and the median number of photographs was 15 (IQR 5-25). Most of those who took photographs did so with the sole intention of presenting them to a physician (38/71, 54%), only 8% (6/71) took the photographs for their own use, and 38% (27/71) took the photographs both for their own use and for the physician. Most of the photographs were of upper limbs (26/71, 37%) or the head and neck (23/71, 32%). Of all evaluated photographs, 85% (60/71) were of good quality, 7% (5/71) were of acceptable quality, and 9% (6/71) were of bad quality based on lighting, resolution, clarity, and focus. In total, 89% (63/71) of the smartphone photographs had the skin lesion in focus, of which 92% (65/71) were sharp and 9% (6/71) were blurred. Most of the smartphone photographs (61/71, 86%) were assessed to be useful for diagnostic and clinical evaluation (Table 3).

**Table 3.** Smartphone photographs taken by the patients coming to consultation in outpatient clinics (n=71).

	Photographs, n (%)
Body region	
Head and neck	23 (32)
Chest and stomach	6 (8)
Back	11 (15)
Upper limb	26 (37)
Lower limb	4 (6)
Missing	1 (1)
Lesion in focus	
Agree	63 (89)
Disagree	8 (11)
Sharp photograph	
Agree	65 (92)
Disagree	6 (9)
Useful in diagnostic evaluation	
Agree	61 (86)
Disagree	10 (14)
Useful in severity assessment	
Agree	59 (83)
Disagree	12 (17)
Resolution	
Good	63 (89)
Acceptable	8 (11)
Bad	0 (0)
Lighting	
Good	61 (86)
Acceptable	4 (6)
Bad	6 (8)
Photo quality	
Good	60 (85)
Acceptable	5 (7)
Bad	6 (8)

For EASI items, induration (14/71, 20%) and lichenification (10/71, 14%) were often difficult to assess (Table 4), and for SCORAD items, lichenification (11/71, 16%) and dryness (13/71, 18%) proved the biggest challenge (Table 5).

**Table 4.** Severity assessment of atopic dermatitis lesion photographs based on Eczema Area and Severity Index (EASI) (n=71).

	EASI score, n (%)				
	None	Mild	Moderate	Severe	Difficult to assess
Erythema	1 (1)	20 (28)	24 (34)	22 (31)	4 (6)
Induration	16 (23)	13 (18)	21 (30)	7 (10)	14 (20)
Excoriation	27 (38)	16 (23)	13 (18)	9 (13)	6 (8)
Lichenification	26 (37)	20 (28)	7 (10)	8 (11)	10 (14)

**Table 5.** Severity assessment of atopic dermatitis lesion photographs based on Scoring Atopic Dermatitis (SCORAD) tool (n=71).

	SCORAD score, n (%)					
	None	Mild	Moderate	Severe	Very severe	Difficult to assess
Erythema	2 (3)	19 (27)	20 (28)	14 (20)	12 (17)	4 (6)
Edema	19 (27)	16 (23)	12 (17)	9 (13)	7 (10)	8 (11)
Oozing	41 (58)	15 (21)	6 (9)	2 (3)	1 (1)	6 (9)
Excoriation	31 (44)	12 (17)	12 (17)	8 (11)	2 (3)	6 (9)
Lichenification	28 (39)	12 (17)	7 (10)	10 (14)	3 (4)	11 (16)
Dryness	15 (21)	19 (27)	11 (16)	9 (13)	4 (6)	13 (18)

### Characteristics of Patients Who Took Smartphone Photographs of Skin Lesions

We found a significant difference in mean age between patients who took photographs and those who did not of 16.3 years (95% CI 8.15-24.46; *P*<.001). The mean age of patients who took smartphone photographs was 29.2 (SD 18.9) years, and that of patients who did not take smartphone photographs was 45.5 (SD 17.8) years. Previous digital contact with the health care system was associated with an increased odds ratio (OR) of 7.19 (95% CI 1.31-39.51; *P*=.01) of taking a skin lesion smartphone photograph. Patients receiving topical monotherapy had a higher chance of taking a skin lesion photograph (OR 4.17, 95% CI 1.42-12.16; *P*=.006), and patients receiving systemic treatment had a lower risk of taking a skin lesion photograph (OR 0.20, 95% CI 0.07-0.59; *P*=.002; Table 6). In logistic regression analysis, use of topical treatment was a statistically significant predictor for the probability of taking a photograph of a skin lesion (OR 5.67, 95% CI 1.20-26.77; β=1.74; SE 0.79; *P*=.03).

**Table 6.** Comparison between patients who took at least 1 smartphone photograph of their skin lesions and those who did not.

Characteristic	Photograph (n=71), n (%)	No photograph (n=29), n (%)	OR^a^ (95% CI)	*P* value
Sex			1.24 (0.50‐3.05)	.64
Male	43 (61)	19 (66)		
Female	28 (39)	10 (34)		
Age (years)			0.18 (0.07-0.49)	<.001
<30	45 (63)	7 (24)		
>30	26 (37)	22 (76)		
Residence			2.03 (0.79-5.21)	.14
Capital region	40 (56)	21 (72)		
Urban area	31 (44)	8 (28)		
Age at disease onset (years)			1.08 (0.45-2.55)	.87
<2	38 (54)	15 (52)		
>2	33 (46)	14 (48)		
Topical treatment only			4.17 (1.42-12.16)	.006
Yes	33 (46)	5 (17)		
No	38 (54)	24 (83)		
Traditional immunosuppressants			0.88 (0.28-2.80)	.83
Yes	11 (15)	5 (17)		
No	60 (85)	24 (83)		
Systemic treatment^b^			0.20 (0.07-0.59)	.002
Yes	35 (49)	24 (83)		
No	36 (51)	5 (17)		
Biologics or JAK^c^ inhibitors			0.25 (0.10-0.63)	.002
Yes	23 (32)	19 (66)		
No	48 (68)	10 (34)		
Preferred remote visit^d^			1.23 (0.42-3.66)	.71
Yes	37 (52)	15 (52)		
No	14 (20)	7 (24)		
Daily use of technology			8.07 (0.80-81.17)	.07
Yes	70 (99)	26 (90)		
No	1 (1)	3 (10)		
Digital contact with the health care system^e^			7.19 (1.31-39.51)	.01
Yes	69 (97)	24 (83)		
No	2 (3)	5 (17)		
Impact of disease on quality of life			0.64 (0.42-0.97)	.04
None	17 (24)	16 (55)		
Small	26 (37)	5 (17)		
Moderate	15 (21)	4 (14)		
Large	6 (8)	4 (14)		
Very large	7 (10)	0 (0)		

a OR: odds ratio.

b Systemic treatment included dupilumab, tralokinumab, baricitinib, abrocitinib, methotrexate, azathioprine, and mycophenolate mofetil.

c JAK: Janus kinase.

d Includes “strongly agree” or “agree” vs “strongly disagree” or “disagree.”

e Includes both digital correspondence with the health care system and digital access to blood samples or medical records.

## Discussion

Hospital outpatients with AD had high digital readiness, with 78% (78/100) using a computer, laptop, or tablet often or always. Almost 3 out of 4 had taken a photograph of their AD skin lesion on their smartphone, mostly with the intention of presenting it to a physician. Furthermore, 85% (60/71) of the photographs were of good quality; however, induration, lichenification, and dryness were often difficult to assess. Receiving topical monotherapy was associated with a higher chance of taking a skin lesion photograph, supporting the demand for tailored monitoring depending on patients’ preferences and risk of flair. AD is very heterogeneous in terms of symptoms, skin manifestations, body area involved, extent, course, and comorbidities. Therefore, it is very unlikely that all patients with AD will respond equally well to treatments. Biomarkers will lead to better identification of patients who will benefit from immunomodulatory treatments, leading to more individualized management [18]. Traditionally, patients on immunosuppressive drugs have often planned consultations in the clinic at certain intervals. Due to better disease control with targeted therapies, these patients only need to be followed up on, for example, once every year; however, due to the expenses related to the treatments, close monitoring will be beneficial for timely drug dose tapering to reduce unnecessary health care expenditures. On the other hand, many patients with mild to moderate disease will still be on traditional immunosuppressive drugs, not meeting the criteria for expensive biological treatments. These patients will often experience flairs in between scheduled consultations. Our study showed that more than half of patients with AD followed up on in an outpatient clinic preferred a remote or online visit instead of an in-person visit at the clinic. Furthermore, there is increasing evidence that patients with skin diseases often take good-quality photographs of their skin lesions with their smartphones [4] and that photographs have high validity and reliability [7,8,19]. This is supported by our findings. Tailored monitoring considering the age, digital readiness, type of treatment, and preferences of the patients may lead to a reduction in health care costs and help pivot consultations toward focused care based on individual needs.

Smartphones are easily accessible and extensively used to take photographs. Many photographs are taken on a daily basis, and more than 90% of all photographs are taken in 2020 using smartphones [20]. Many people find it natural to take photographs for memory or documentation [20]; hence, taking photographs of skin lesions is widely practiced [4]. There is a demand for integrating smartphone photographs into clinical practice to assess disease fluctuation in between physical examinations. Educating patients in how to take a good clinical photograph of AD skin lesions may improve the quality and utility of the photographs in a clinical setting. Information regarding distance between the camera and the skin lesion (approximately 20 cm), using a uniform background, and taking the photograph in good natural lighting is especially important. Furthermore, using photographs in a clinical setting through a remote visit to replace a physical consultation requires thorough patient education in the assessment of body surface area and selection of representative lesions in each anatomical area included in the EASI or SCORAD.

Even though the task of evaluating the quality of photographs was clearly defined to create consistency in evaluations, this study was limited by a lack of multiple raters to evaluate the same photograph due to logistical challenges in a clinical survey.

In conclusion, patients with AD followed up on in an outpatient clinic often took high-quality photographs of their skin lesions before consultations with the intention of presenting them to the physicians. More evidence for tailored or personalized monitoring through remote visits using photographs of skin lesions and its effect on health care costs is warranted.

## Abbreviations

AD: atopic dermatitis
EASI: Eczema Area and Severity Index
OR: odds ratio
SCORAD: Scoring Atopic Dermatitis
